# The Effectiveness of Polyhydroxyalkanoate (PHA) Extraction Methods in Gram-Negative *Pseudomonas putida* U

**DOI:** 10.3390/polym17020150

**Published:** 2025-01-09

**Authors:** Luis Getino, Irene García, Alfonso Cornejo, Raúl Mateos, Luisa M. Ariza-Carmona, Natalia Sánchez-Castro, José F. Moran, Elías R. Olivera, Alejandro Chamizo-Ampudia

**Affiliations:** 1 Área de Bioquímica y Biología Molecular, Departamento de Biología Molecular, Universidad de León, 24007 León, Spain; luis.getino@unileon.es (L.G.); erodo@unileon.es (E.R.O.); 2 IES Los Sauces—Avda Federico Silva, 48, Benavente, 49600 Zamora, Spain; irene.garrom.1@educa.jcyl.es; 3 Institute for Advanced Materials and Mathematics (INAMAT2), Department of Sciences, Public University of Navarre (UPNA), Campus de Arrosadía, 31006 Pamplona, Spain; alfonso.cornejo@unavarra.es; 4 Chemical and Environmental Bioprocess Engineering Group, I4 Institute, University of León, 24071 León, Spain; rmatg@unileon.es; 5 Faculty of Education, International University of La Rioja, 26006 Logroño, Spain; luisamaria.ariza@unir.net; 6 Manchester Institute of Biotechnology, University of Manchester, Manchester M13 9PL, UK; natalia.sanchezcastro@postgrad.manchester.ac.uk; 7 Institute for Multidisciplinary Research in Applied Biology (IMAB), Department of Sciences, Public University of Navarre (UPNA), Avda. de Pamplona 123, 31192 Mutilva, Spain; jose.moran@unavarra.es; 8 Instituto de Biología Molecular, Genómica y Proteómica (INBIOMIC), Universidad de León, Campus de Vegazana, 24071 León, Spain

**Keywords:** *Pseudomonas putida* U, extraction processes, polyhydroxyalkanoates, environmentally friendly solvents, purification

## Abstract

Bioplastics are emerging as a promising solution to reduce pollution caused by petroleum-based plastics. Among them, polyhydroxyalkanoates (PHAs) stand out as viable biotechnological alternatives, though their commercialization is limited by expensive downstream processes. Traditional PHA extraction methods often involve toxic solvents and high energy consumption, underscoring the need for more sustainable approaches. This study evaluated physical and chemical methods to extract PHAs from *Pseudomonas putida* U, a bacterium known to produce poly-3-hydroxyoctanoate P(3HO). Lyophilized cells underwent six extraction methods, including the use of the following: boiling, sonication, sodium hypochlorite (NaClO), sodium dodecyl sulfate (SDS), sodium hydroxide (NaOH), and chloroform. Physical methods such as boiling and sonication achieved yields of 70% and 60%, respectively, but P(3HO) recovery remained low (30–40%). NaClO extraction provided higher yields (80%) but resulted in significant impurities (70%). NaOH methods offered moderate yields (50–80%), with P(3HO) purities between 50% and 70%, depending on the conditions. Spectroscopic and analytical techniques (FTIR, TGA, NMR, GPC) identified 0.05 M NaOH at 60 °C as the optimal extraction condition, delivering high P(3HO) purity while minimizing environmental impact. This positions NaOH as a sustainable alternative to traditional halogenated solvents, paving the way for more eco-friendly PHA production processes.

## 1. Introduction

Due to their diverse applications, plastics are considered essential in modern life [1]. In 2022, 390.7 Mt of plastic were manufactured globally, with 90.2% being derived from fossil fuels. In comparison, only 1.51% had a bio-based origin, and only 8.32% was sourced from recycled materials [2]. Both plastic production and disposal are known to significantly impact the environment. The extraction of raw precursors, their transportation, polymer manufacturing, and their final distributions generate substantial greenhouse gas emissions [3]. Moreover, due to their recalcitrant nature and the fact that only 13–15% of the plastic produced annually is recycled, the remainder often ends up in landfills or oceans [4,5]. These plastic wastes degrade into microplastics, which, when ingested by wildlife, enter the food chain, raising concerns about their potential link to pathological processes in animals and humans [6].

In response to these issues, the use of more sustainable and biodegradable materials has been proposed [7]. Bioplastics have emerged as a promising alternative [8,9,10]. These are manufactured from renewable resources rather than fossil fuels, with processes that emit up to 80% less carbon dioxide (CO_2_) compared to petrochemical plastics [9]. Bioplastics can be either biodegradable, such as polyhydroxyalkanoates (PHAs), polysaccharide-based plastics, polylactic acid (PLA), polybutylene adipate-co-terephthalate (PBAT), and polyvinyl alcohol (PVA) [7], or non-biodegradable, such as bio-polyethylene (Bio-PE), bio-polypropylene (Bio-PP), bio-polyethylene terephthalate (Bio-PET), or bio-polyethylene furanoate. Biodegradability is determined by the compound’s chemical structure, not the raw materials used for its production [9].

Bioplastics can be synthesized from various sources, including molasses, industrial wastes, and petroleum byproducts [10,11,12,13,14]. Polymers such as starch or cellulose are also suitable for bioplastic production [15,16]. According to their precursors, bioplastics are classified into the following: (I) agro-polymers derived from natural sources like starch or cellulose; (II) biomaterials synthesized from renewable raw materials such as lactic acid, lipids, proteins, or polysaccharides to produce polylactic acid, bio-nylons, or bio-polyurethanes; and (III) biopolymers obtained through microbial activity, including PHAs, cellulose, or polyphosphates [16,17,18].

PHAs, due to their biological origin, degrade into CO_2_ and water under both aerobic and anaerobic conditions, enabling their recycling [19]. These biopolyesters are categorized based on the number of carbons in their monomers: short-chain-length PHAs (scl-PHAs) with 3–5 carbons, medium-chain-length PHAs (mcl-PHAs) with 6–14 carbons, and long-chain-length PHAs (lcl-PHAs) with 15 or more carbons [20,21,22,23]. PHAs are accumulated as intracellular granules in many bacteria, consisting of a polymeric core surrounded by a surface layer of proteins and lipids that varies by species [24]. Some *Pseudomonas* strains are capable of accumulating PHAs until they make up to 90% of their dry weight [25,26].

One of the main factors contributing to the high cost of PHAs compared to petroleum-based plastics is the extraction process, which accounts for 30% of production costs [1,21,22]. Extraction methods are classified into chemical and physical techniques, applied either individually or in combination. Typically, these processes begin with biomass drying, and culminate in polymer purification [27]. Some treatments, such as sodium hypochlorite (NaClO), bypass the drying step. Chemical methods involving organic solvents achieve high extraction efficiencies while preserving the polymer and removing endotoxins [28,29,30]. However, these processes often rely on toxic, non-recyclable solvents like chloroform (CHCl_3_), which are polluting, expensive, and harmful to health. More sustainable alternatives, including hydrochloric acid (HCl), NaClO, sodium hydroxide (NaOH), acetic acid (CH_3_COOH), acetone (C_3_H_6_O), hexane (C_6_H_14_), and surfactants like sodium dodecyl sulfate (SDS), have been proposed [10,31,32,33,34].

Enzymatic methods, utilizing proteases or glucosidases, offer a greener approach to PHA recovery, but are limited by high costs during production and their specificity to the bioplastic or microorganism used [10,35,36]. Physical methods such as sonication, microwave treatment, agitation, gamma radiation, or ball milling have also been explored [10,36,37,38,39,40,41]. Among these, sonication has shown high efficiency, although its effectiveness depends on factors such as pH, sonication source, and combinations with solvents or surfactants [10,37,40].

**Figure 1:**
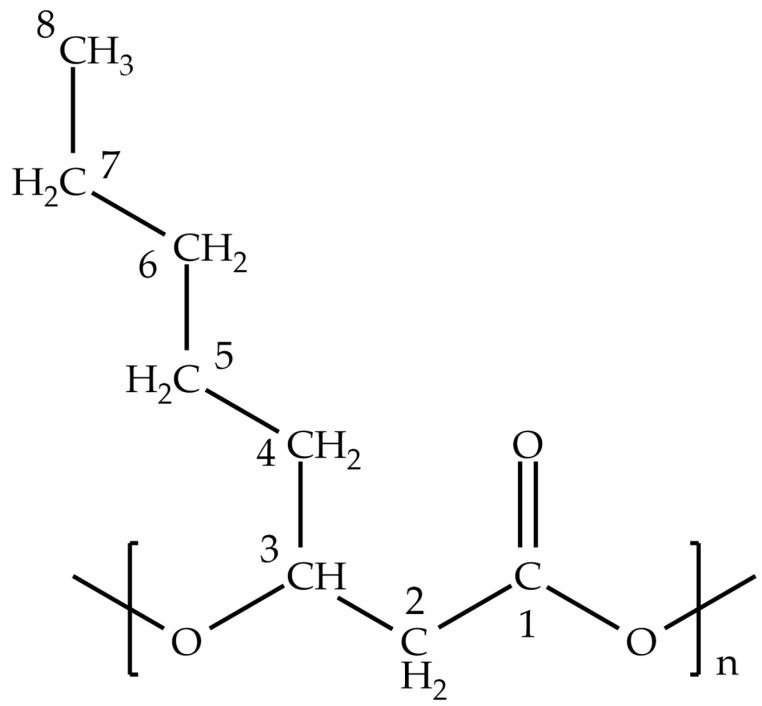
General structure of the poly-3-hydroxyoctanoate P(3HO) produced by *P. putida* U.

To improve the sustainability of PHA production, more environmentally friendly extraction reagents must be adopted [42]. This study investigated the recovery of mcl-PHAs from the Gram-negative bacterium *Pseudomonas putida* U, which is known to produce poly-3-hydroxyoctanoate P(3HO) (Figure 1) [43,44]. Several established and novel extraction methods were used to recover the polyhydroxyalkanoate. Yields and purities were compared with those obtained using chloroform, one of the most commonly used extraction solvents, through various characterization techniques.

## 2. Materials and Methods

### 2.1. Reagents

Biochemical reagents, including culture media, carbon sources, and antibiotics, were obtained from Condalab (Madrid, Spain) and Sigma-Aldrich (St. Louis, MO, USA). Chemical reagents used for PHA extraction—chloroform (CAS 67-66-3), NaOH (CAS 1310-73-2), SDS (CAS 151-21-3), and NaClO (CAS 7681-52-9)—were supplied by VWR (Radnor, PA, USA) and Thermo Scientific (Waltham, MA, USA).

### 2.2. Strains and Growing Conditions

The strain derived from *P. putida* U, which lacks beta-oxidation genes (*fadBA*), hereafter referred to as ΔfadBA, was used for its ability to accumulate large amounts of PHA internally [26,43]. This strain was maintained by routine culturing on LB-agar plates supplemented with rifampicin (100 µg/mL).

For PHA accumulation, minimal medium for *Pseudomonas* (MM) was used [26,45], containing 13.6 g/L KH_2_PO_4_, 0.25 g/L MgSO_4_·7H_2_O, 2 g/L (NH_4_)_2_SO_4_, and 0.5 mg/L FeSO_4_·7H_2_O, supplemented with 10 mM 4-hydroxyphenylacetic acid (4-OH-PhAc) as the carbon and energy source, and 10 mM octanoic acid as a P(3HO) precursor. A pre-inoculum of ΔfadBA was prepared by inoculating at OD540 = 0.05 in MM supplemented with 10 mM 4-OH-PhAc and rifampicin (25 µg/mL). Incubations were carried out in a rotary shaker (250 rpm) at 30 °C. After 14 h of growth, cells were collected by centrifugation (5800× *g*; 8 min; 6 °C). The supernatant was removed, and the bacterial pellets were stored at −80 °C until use.

### 2.3. Dehydration of Samples

To ensure complete drying of the samples, aliquots of approximately 400 mg of bacterial biomass were transferred into 1.5 mL Eppendorf tubes. The drying process was then performed using a lyophilizer (Telstar Cryodos, Terrassa, Spain) for a duration of 20 h [42].

### 2.4. Extraction Methods

Among the PHA extraction methods employed in this work, six chemical and physical techniques were used. All methods began with the same homogeneous mixture of bacteria, which was dried in an oven at 45 °C. The procedures for each method are as follows:

**Method 1: Boiling.** Approximately 400 mg of dried biomass was resuspended in 10 mL of sterile distilled water and boiled for 1 h. After incubation, the samples were allowed to reach room temperature and were centrifuged (20 °C; 5 min; 3220× *g*). The supernatant was removed, and the pellet was washed twice with 5 mL of distilled water. The centrifuged pellet was dried in an oven at 45 °C for 24 h before weighing.

**Method 2: Physical Decomposition** [37]. Approximately 400 mg of biomass was resuspended in 16 mL of sterile distilled water. Ten pulses of 30 s each, with 1 min pauses in between, were performed using a digital sonicator (Branson, Sonifier 450, power 300 W, Ridge Road, Brookfield, CT, USA) at 70% amplitude [46]. After sonication, the sample was transferred to another container using an additional 4 mL of sterile distilled water to ensure complete collection. The tubes were centrifuged for 30 min at 3220× *g* and room temperature, and the supernatant was removed. Finally, the pellets were placed in an oven at 45 °C for 24 h before weighing.

**Method 3: Sodium Hypochlorite (NaClO)** [33]. Aliquots of approximately 400 mg of biomass were resuspended in 33.8 mL of NaClO (3.7% Cl_2_). The samples were incubated for 5, 15, and 60 min at 25 °C and 90 °C. After incubation, the samples were cooled to room temperature (20 °C) and the extraction products were filtered (0.45 µm, cellulose) to remove the aqueous phase. The filter, along with the residue, was placed in an oven to remove residual water. The centrifuged pellet (20 °C; 20 min; 9000× *g*) was oven-dried at 45 °C for 24 h before weighing.

**Method 4: Sodium Dodecyl Sulfate (SDS)** [47]. Approximately 400 mg of dried biomass was added to 24 mL of a 3.33% (*w*/*v*) SDS solution at 90 °C. The samples were incubated for 3 h and mixed every 30 min. Once room temperature (20 °C) was reached, the extraction products were filtered to remove the liquid phase. The solid residues, along with the filter, were then placed in the oven. The centrifuged pellet (20 °C; 5 min; 3220× *g*) was dried in an oven at 45 °C for 24 h before weighing.

**Method 5: Alkaline Lysis (NaOH)** [31]. The dried biomass (approximately 400 mg) was resuspended in 20 mL of a sodium hydroxide solution (NaOH) (0.01 M; 0.05 M; and 0.1 M) and incubated for 1 or 5 h at temperatures of 30 °C or 60 °C. After incubation, the samples were centrifuged several times with distilled water between centrifugations (9000× *g*; 20 °C, 5 min). The residue was transferred to an oven to remove residual water. The centrifuged pellet was oven-dried at 45 °C for 24 h before weighing.

**Method 6: Chloroform** [29]. Approximately 400 mg of dried biomass was placed in a previously tared round-bottom flask and resuspended in 50 mL of chloroform. A reflux system was used to boil the samples for 2 h. After completion, a rotary evaporator was employed to remove almost all organic solvents. The organic solvent was filtered off and transferred to 60 mm glass Petri dishes, which were placed in an oven at 45 °C to evaporate the chloroform for 24 h.

### 2.5. PHA Purification

The solids from each extraction were resuspended in 10 mL of chloroform and mixed vigorously for approximately 10 min (except for the samples initially extracted with chloroform). The organic solvent was then filtered and transferred to 60 mm glass Petri dishes, which were placed in an oven at 45 °C to evaporate all the chloroform for 24 h [48].

### 2.6. Fourier Transform Infrared Spectroscopy (FT-IR)

The prepared films were analyzed using attenuated total reflection infrared (ATR) spectroscopy. The samples were pressed against a diamond, which is commonly used as the internal reflective element (IRE), with a constant torque of 0.22 Nm, using a torque spanner. Two hundred scans with a resolution of 4 cm^−1^ were acquired from 4000 cm^−1^ to 400 cm^−1^ on a Jasco FT/IR-4700 Fourier transform infrared spectrophotometer (Tokyo, Japan). The spectra were obtained by subtracting the baseline spectrum from a clean IRE. The wavelengths at which the bands appeared in the ATR spectra were used to identify the composition and purity of the PHA films, and the intensities of the bands indicated changes in crystallinity relative to the depth of penetration.

### 2.7. Thermogravimetric Analysis (TGA)

The thermal degradation of the PHAs obtained by the different extraction methods was determined using a TGA SDT 650 system (TA Instruments, New Castle, DE, USA). TGA scans were performed from 25 °C to 380 °C on 5–8 mg samples, at a heating rate of 10 °C/min with a nitrogen flow rate of 100 mL/min.

### 2.8. Gel Permeation Chromatography (GPC)

Gel permeation chromatography measurements were carried out in an Agilent 1100 (Santa Clara, CA, USA) equipped with an UV-VIS absorption detector at 254 nm coupled with a refractive index detector (RID) using coupled HR-5 and HR-1 Styragel columns (Waters, Milford, MA, USA) at 30 °C as the stationary phase and THF as the mobile phase at 1 mL min^−1^ flow. 10 mg of sample were dissolved in 1 mL of THF and filtered through a 0.45 mm PTFE filter prior to injection of 20 μL of this solution.

The number-average molecular weight (Mn), weight-average molecular weight (Mw), and polydispersity index (PDI) of the polymers were determined by GPC. The PDI, which measures the molecular mass distribution in each polymer sample, was calculated as the ratio of the weight-average molecular weight (Mw) to the number-average molecular weight (Mn).

### 2.9. ^13^C and ^1^H NMR

Nuclear magnetic resonance (NMR) measurements were conducted at 300 K in a Bruker Ascend III spectrometer equipped with a PH BBI 400S1 probe (Bruker Co., Billerica, MA, USA), at 400 MHz and 101 MHz for ^1^H and ^13^C respectively, and were processed using Bruker Topspin 3.6.2 and Dynamics Center 2.6.1 software. ^1^H and ^13^C experiments were referenced using the residual signal for CDCl_3_ at 7.26 ppm and 77.1 ppm, respectively. ^1^H experiments were run using the *zg30* pulse program at 16 scans. ^13^C experiments were conducted using the *jmod* pulse program at 3500 scans.

**Sonication**: ^1^H (CDCl_3_, d-ppm): 5.17 (quintuplet, J = 6.3 Hz, *1H*, H_3_), 2.56 (dd, J = 15.4, 6.7 Hz, *1H*, H_2_), 2.48 (dd, J = 15.4, 5.9 Hz, *1H*, H_2_′), 1.63–1.51 (m, *2H*, H_4_), 1.36–1.19 (m, *3H*, H_5_, H_6_, H_7_), 0.87 (t, J = 6.6 Hz, 3H, H_8_).
  ^13^C (CDCl_3_, d-ppm): 169.5 (C_1_), 71.0 (C_3_), 39.2 (C_2_), 33.9 (C_4_), 31.6 (C_5_), 24.8 (C_6_), 22.6 (C_7_), 14.1 (C_8_).
**Chloroform**: ^1^H (CDCl_3_, d-ppm): 5.17 (quintuplet, J = 6.5 Hz, *1H*, H_3_), 2.56 (dd, J = 15.3, 6.9 Hz, *1H*, H_2_), 2.48 (dd, J = 15.3, 5.7 Hz, *1H*, H_2_′), 1.64–1.50 (m, *2H*, H_4_), 1.36–1.18 (m, *3H*, H_5_, H_6_, H_7_), 0.87 (t, J = 5.9 Hz, *3H*, H_8_).
  ^13^C (CDCl_3_, d-ppm): 169.5 (C_1_), 71.0 (C_3_), 39.2 (C_2_), 33.9 (C_4_), 31.6 (C_5_), 24.8 (C_6_), 22.6 (C_7_), 14.1 (C_8_).
**NaOH, 0.01 M, 60 °C, 1 h**: ^1^H (CDCl_3_, d-ppm): 5.17 (quintuplet, J = 6.3 Hz, *1H*, H_3_), 2.56 (dd, J = 15.4, 7.0 Hz, *1H*, H_2_), 2.48 (dd, J = 15.4, 5.9 Hz, *1H*, H_2_′), 1.63–1.51 (m, *2H*, H_4_), 1.35–1.20 (m, *3H*, H_5_, H_6_, H_7_), 0.86 (t, J = 6.5 Hz, *3H*, H_8_).
  ^13^C (CDCl_3_, d-ppm): 169.5 (C_1_), 71.0 (C_3_), 39.2 (C_2_), 33.9 (C_4_), 31.6 (C_5_), 24.8 (C_6_), 22.6 (C_7_), 14.1 (C_8_).
**NaOH, 0.05 M, 60 °C, 1 h**: ^1^H (CDCl_3_, d-ppm): 5.17 (quintuplet, J = 6.3 Hz, *1H*, H_3_), 2.56 (dd, J = 15.4, 7.0 Hz, *1H*, H_2_), 2.48 (dd, J = 15.4, 5.9 Hz, *1H*, H_2_′), 1.63–1.51 (m, *2H*, H_4_), 1.33–1.20 (m, *3H*, H_5_, H_6_, H_7_), 0.86 (t, J = 6.6 Hz, 3H, H_8_).
  ^13^C (CDCl_3_, d-ppm): 169.5 (C_1_), 71.0 (C_3_), 39.2 (C_2_), 33.9 (C_4_), 31.6 (C_5_), 24.8 (C_6_), 22.6 (C_7_), 14.1 (C_8_).
**NaOH, 0.1 M, 60 °C, 1 h**: ^1^H (CDCl_3_, d-ppm): 5.17 (quintuplet, J = 6.5 Hz, *1H*, H_3_), 2.56 (dd, J = 15.4, 7.0 Hz, *1H*, H_2_), 2.49 (dd, J = 15.4, 5.9 Hz, *1H*, H_2_′), 1.63–1.50 (m, *2H*, H_4_), 1.33–1.17 (m, *3H*, H_5_, H_6_, H_7_), 0.86 (t, J = 6.4 Hz, 3H, H_8_).
  ^13^C (CDCl_3_, d-ppm): 169.5 (C_1_), 71.0 (C_3_), 39.2 (C_2_), 33.9 (C_4_), 31.6 (C_5_), 24.8 (C_6_), 22.6 (C_7_), 14.1 (C_8_).
**Boiling**: ^1^H (CDCl_3_, d-ppm): 5.16 (quintuplet, J = 6.3 Hz, *1H*, H_3_), 2.56 (dd, J = 15.4, 7.0 Hz, *1H*, H_2_), 2.49 (dd, J = 15.4, 6.2 Hz, *1H*, H_2_′), 1.63–1.51 (m, *2H*, H_4_), 1.33–1.20 (m, *3H*, H_5_, H_6_, H_7_), 0.87 (t, J = 5.3 Hz, *3H*, H_8_).
  ^13^C (CDCl_3_, d-ppm): 169.6 (C_1_), 71.0 (C_3_), 39.2 (C_2_), 33.9 (C_4_), 31.6 (C_5_), 24.8 (C_6_), 22.6 (C_7_), 14.1 (C_8_).
**SDS**: ^1^H (CDCl_3_, d-ppm): 5.17 (quintuplet, J = 6.3 Hz, *1H*, H_3_), 2.55 (dd, J = 15.4, 7.0 Hz, *1H*, H_2_), 2.49 (dd, J = 15.4, 5.9 Hz, *1H*, H_2_′), 1.63–1.51 (m, *2H*, H_4_), 1.33–1.18 (m, *3H*, H_5_, H_6_, H_7_), 0.86 (t, J = 6.4 Hz, 3H, H_8_).
  ^13^C (CDCl_3_, d-ppm): 169.5 (C_1_), 71.0 (C_3_), 39.2 (C_2_), 33.9 (C_4_), 31.6 (C_5_), 24.8 (C_6_), 22.6 (C_7_), 14.1 (C_8_).
**NaClO, 60′**: ^1^H (CDCl_3_, d-ppm): 5.17 (quintuplet, J = 6.3 Hz, *1H*, H_3_), 2.55 (dd, J = 15.3, 7.0 Hz, *1H*, H_2_), 2.49 (dd, J = 15.4, 5.9 Hz, *1H*, H_2_′), 1.63–1.51 (m, *2H*, H_4_), 1.35–1.19 (m, *3H*, H_5_, H_6_, H_7_), 0.87 (t, J = 6.6 Hz, *3H*, H_8_).
  ^13^C (CDCl_3_, d-ppm): 169.5 (C_1_), 70.9 (C_3_), 39.2 (C_2_), 33.8 (C_4_), 31.6 (C_5_), 24.8 (C_6_), 22.6 (C_7_), 14.1 (C_8_).

### 2.10. Analytical Software and Mathematical Models

Statistical analyses were conducted using GraphPad Prism 6 (San Diego, CA, USA) to evaluate the significance of the observed differences. An ANOVA test, followed by Tukey’s post hoc test, was performed for all conditions, with significance set at *p* < 0.05.

For each extraction process, the percentage of dry biomass obtained was calculated relative to the initial wet biomass:

(1) $${\%}_{\text{Dry biomass}}=\frac{\text{Dry biomass weight}}{\text{Wet biomass weight}}\times 100$$

For each extraction process the percentage of raw PHA extracted was calculated relative to the initial dry biomass.

(2) $${\%}_{\text{Raw PHA}}=\frac{\text{Raw PHA weight}}{\text{Dry biomass weight}}\times 100$$

Finally, after purification, the percentage of PHAs present in each extract was calculated as described below.

(3) $${\%}_{\text{PHA}}=\frac{\text{PHA weight}}{\text{Raw PHA weight}}\times 100$$

## 3. Results

### 3.1. Extraction Process of PHAs

*P. putida* strain ΔfadBA was cultured in a minimal medium containing 10 mM4-OH-PhAc as a carbon and energy source and 5 mM octanoic acid as a precursor for P(3HO) accumulation. The resulting biomass was subjected to a drying process through lyophilization. After drying, aliquots of 400 mg were taken and treated using the different extraction methods described in the Materials and Methods section.

**Figure 2:**
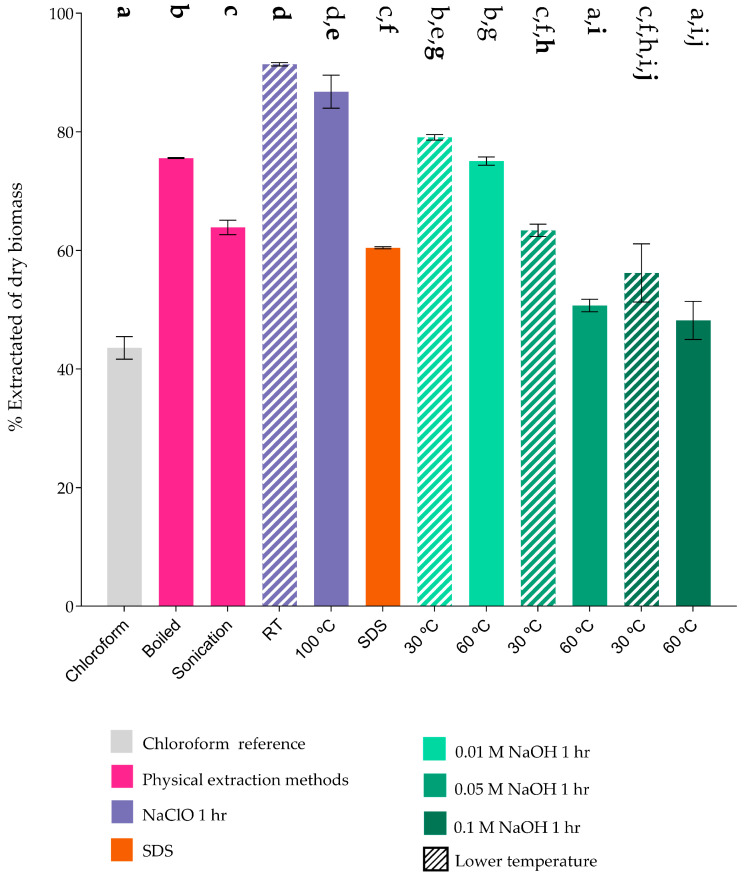
Extraction of PHAs by various methods. The chloroform (gray) reference was compared to physical extraction (magenta), NaClO (purple), SDS (orange), and NaOH (green) methods. All methods except 0.05 M NaOH at 30 °C for 5 h (Figure S1), 0.05 M NaOH at 60 °C for 1 h, and 0.1 M NaOH at 60 °C for 5 h (Figure S1) had a significant difference in the amount of extract obtained with respect to the chloroform control. All samples were run in triplicate and statistical significance was determined with ANOVA between samples (*p* ≤ 0.05). The letters present in multiple columns indicate that no significant difference exists compared to treatments with the same bolded letter.

After the different extraction processes, the different samples were centrifuged to separate the aqueous phase, containing soluble compounds, from the solid phase, which retained the PHAs. The resulting pellet was dried at 45 °C and weighed using a precision balance to quantify the percentage of the extract obtained. This extraction yield was compared with that obtained using chloroform, a technique widely reported in the literature for its high efficiency in PHA recovery [27]. It should be noted that extraction methods involving NaOH and NaClO were tested at various concentrations and temperatures (Figure 2) [49].

The crude PHA extracted from samples treated with NaClO could not be effectively separated by centrifugation, likely due to partial degradation and the formation of a sticky polymer. In this case, the aqueous phase was removed by filtration (0.22 μm), and the remaining filter residue was dried. As shown in Figure 2, the highest extraction values were obtained using NaClO, followed by samples treated with NaOH as the chemical agent. The physical methods reported the lowest extraction values overall.

### 3.2. Quantification Process of Impurities in Extraction

**Figure 3:**
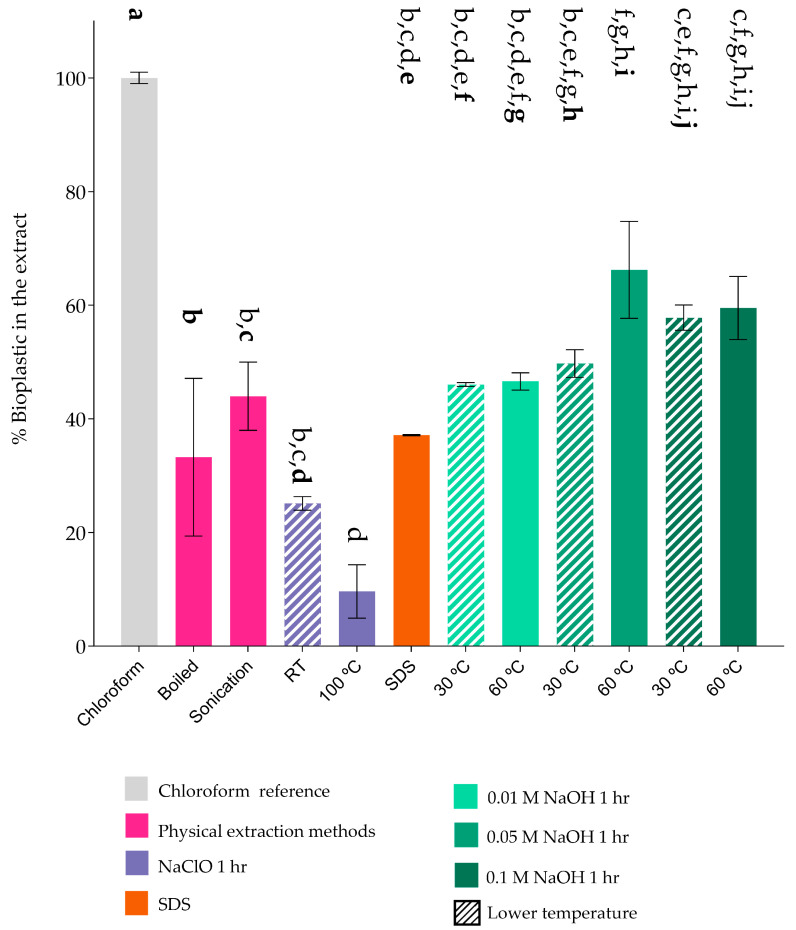
Purity of PHAs obtained by different extraction methods. An extraction using chloroform (gray) was used as a reference to compare the results from the other methods. In magenta the physical treatments are shown, in purple the hypochlorite treatments, in orange the SDS treatment, and finally in green the NaOH treatments. The processes at different times such as NaOH and NaClO can be seen in Figure S2. All extraction processes had significant differences with respect to chloroform. Samples were run in triplicate (n = 3). Statistical significance was determined with ANOVA between samples, with respect to chloroform treatment, and non-significance was considered as (*p* ≤ 0.05). The letters present in multiple columns indicate that no significant difference exists compared to the treatment with the same bolded letter.

To assess the actual amount of PHA extracted by each method, the extracts were resuspended in chloroform to dissolve the PHAs and were compared with the chloroform extraction process. The mixture was filtered, and all the organic solvent was evaporated in an oven at 45 °C for 20 h, as described in the Materials and Methods section. The purity percentage was calculated accordingly (Figure 3).

In some extraction processes, the extracts contained particles, likely polar impurities, which were insoluble in chloroform. These impurities were removed through filtration during the purification method, further reducing the weight of the extracts. Consequently, an initial decrease in the PHA percentage was observed, indicating the presence of polar compounds in the crude PHA. This loss in yield was relative to that obtained through the chloroform extraction process.

### 3.3. Fourier Transform Infrared Spectroscopy (FT-IR) Analysis of PHAs

After obtaining PHAs through various extraction methods, the PHA films were analyzed to assess their purity. Characterization was performed using Fourier Transform Infrared Spectroscopy (FT-IR) and Thermogravimetric Analysis (TGA). Samples extracted using chloroform, boiling, or sodium hypochlorite (NaClO) at room temperature for 60 min, 0.1 M NaOH at 60 °C for 1 h, and SDS were examined.

**Figure 4:**
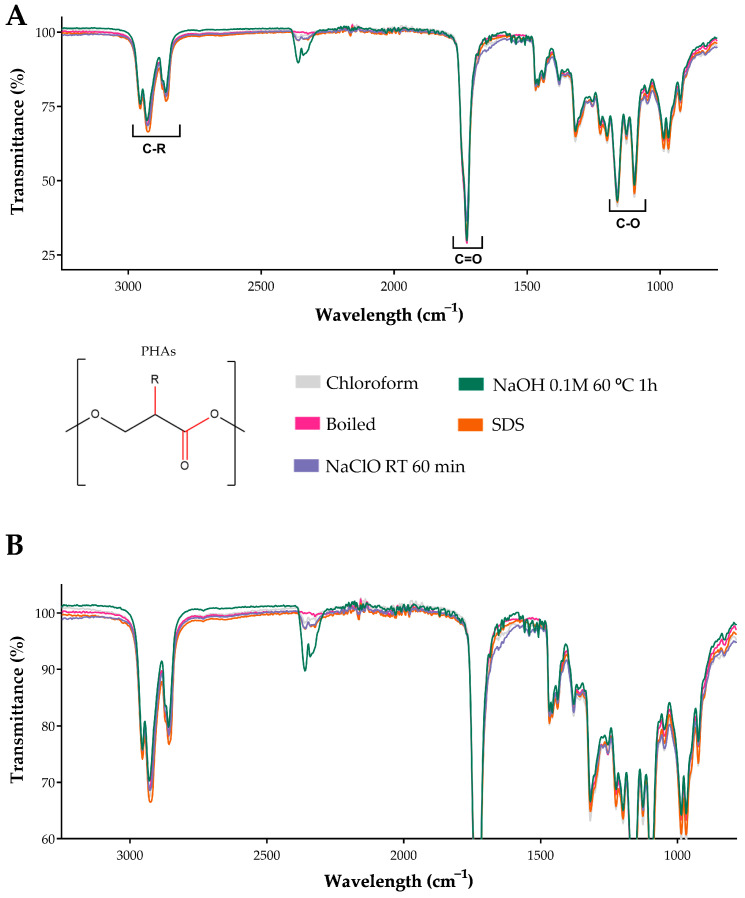
Fourier Transform Infrared Spectroscopy (FT-IR) of the PHAs obtained by the different extraction processes. (**A**) The lines indicate the vibrational frequency of each of the bonds between atoms in the monomer of PHAs. (**B**) Enlargement of the FT-IR spectrum of PHAs. The other samples obtained from the extractions analyzed with FT-IR are shown in Figure S3.

The infrared spectra obtained from each PHA extraction method, analyzed in triplicate, showed nearly 100% overlap with the chloroform extraction reference spectrum, confirming successful PHA extraction across all methods. The only notable difference was observed in the wavelength range of 2500–2300 cm^−1^, where background impurities, such as the presence of CO_2_, were detected. Other characteristic peaks corresponding to ester bonds displayed high similarity across all extracts. Specifically, the carbonyl (C=O) bonds were identified in the 1800–1700 cm^−1^ range, while carbon-oxygen (C–O) bonds were detected at 1300–1000 cm^−1^. Additionally, regardless of the extraction method, all PHAs exhibited vibrational frequencies associated with carbon–hydrogen (C–H) bonds between 3000 and 2700 cm^−1^. These results are illustrated in Figure 4, highlighting the consistency of structural features among PHAs extracted using different processes.

### 3.4. Thermogravimetric Analysis (TGA) of PHAs

**Figure 5:**
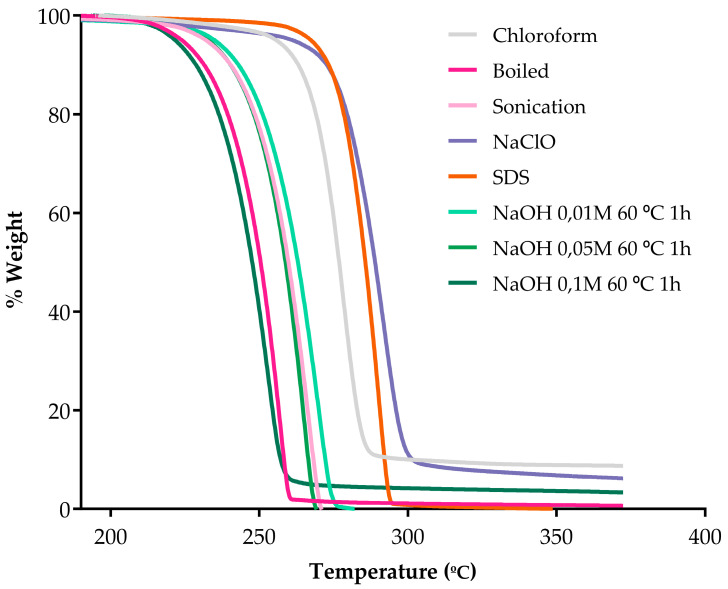
Thermogravimetric Analysis of the different PHA samples. The curves show the thermal degradation of the PHA extracted as indicated.

The thermal purity analysis of PHAs by TGA revealed that samples obtained through boiling and extraction with higher NaOH concentrations thermally degraded at lower temperatures compared to others, within the range of 220–260 °C, with a maximum degradation rate around 255 °C (Figure 5). Higher-purity products exhibited greater homogeneity and therefore evaporated within narrower temperature intervals, resulting in steeper degradation slopes.

In contrast, samples extracted using SDS and NaClO displayed higher thermal resistance, degrading at higher temperatures within the range of 260–300 °C, with a maximum degradation rate near 290 °C. By 380 °C, all samples were completely volatilized except those obtained through boiling, 0.1 M NaOH, NaClO, and chloroform, which left residues of 0.7%, 3.3%, 6.2%, and 8.7%, respectively [50]. Interestingly, the chloroform-extracted sample exhibited the highest residual mass after degradation. These residues, identified through TGA, were heated to 500 °C to assess the stability of impurities, and volatilization was observed (Table S1).

### 3.5. Gel Permeation Chromatography (GPC) Analysis of PHAs

The polymers obtained from the different extraction processes were characterized using gel permeation chromatography (GPC). GPC was employed to determine the weight-average molecular weight (Mw), number-average molecular weight (Mn), and polymer dispersity index (PDI) of each sample after purification by reprecipitation in methanol from a tetrahydrofuran (THF) solution (Table 1).

**Figure 6:**
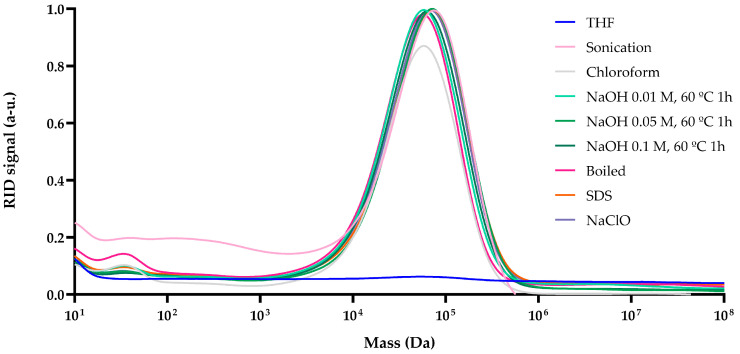
Gel permeation chromatography (GPC) chromatograms of P3HO polymers extracted from *P. putida* U using different extraction methods.

The results indicated that the extraction processes afforded polymers with similar molecular sizes, likely due to the bacterial accumulation of P(3HO) under the given conditions [51]. However, slight differences in molecular weight were observed, potentially attributable to the extraction treatments. Samples treated with 0.01 M NaOH exhibited molecular weights close to those extracted with chloroform (125,312 Da), whereas treatments with 0.05 M and 0.1 M NaOH, SDS, NaClO, and sonication yielded slightly higher molecular weights. While most samples displayed similar molecular weights, notable distinctions in PDI were identified among the extraction methods. The lowest PDI was observed in samples extracted via boiling, with similar values recorded for chloroform and NaOH-based extractions (Table 1). Importantly, only one size distribution was found for each sample and no impurities were detected (Figure 6).

### 3.6. Nuclear Magnetic Resonance (NMR) Analysis of Hydrogen (^1^H) and Carbon (^13^C)

**Figure 7:**
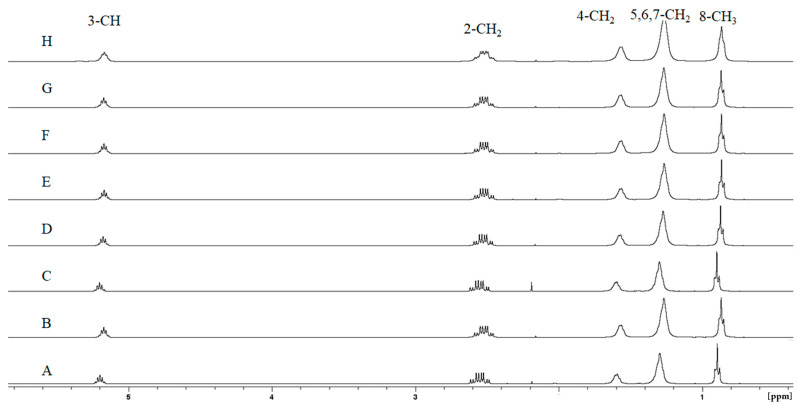
NMR spectra of the P3HO homopolymer extracted using various methods from *P. putida* U. The ^1^H NMR spectra for the extraction methods are presented below: (**A**) boiling, (**B**) sonication, (**C**) SDS, (**D**) NaClO, (**E**) 0.01 M NaOH 60 °C 1 h, (**F**) 0.05 M NaOH 60 °C 1 h, (**G**) 0.1 M NaOH 60 °C 1 h, (**H**) chloroform. Peaks were assigned to the corresponding hydrogen positions of octanoate, as depicted in Figure 1. Peaks around 2.5 ppm (2.56 and 2.48 ppm) were observed, corresponding to the hydrogens on carbon 2. These peaks result from two diastereotopic hydrogens on that carbon, generating slightly different signals in the ^1^H NMR spectrum.

Structural confirmation of the extracted polymer was performed using ^1^H and ^13^C NMR spectroscopy. Figure 7 and Figure S4 present the ^1^H and ^13^C NMR spectra along with the corresponding assignments for the identified PHA. Monomer identification was conducted through direct interpretation and comparison with known P(3HO) NMR spectra [44].

In the ^1^H NMR spectrum (Figure 7), five distinct peaks were observed, corresponding to five unique proton environments within the molecule. The analysis indicated the presence of hydrogen atoms attached to C2 (CH_2_ group), C3 (CH group), C4 (CH_2_ group), C5, C6, C7 (CH_2_ groups), and C8 (CH_3_ group), with chemical shifts of 2.5, 5.2, 1.6, 1.2, and 0.8 ppm, respectively.

In the ^13^C NMR spectrum (Figure S4), eight distinct peaks were detected, representing eight unique carbon environments in the molecule. The chemical shift at 169.52 ppm was attributed to C1 (C=O group), a quaternary carbon, and C3 (CH group), a tertiary carbon, which exhibited a chemical shift at 70.96 ppm. C2 (CH_2_ group), a secondary carbon, displayed a chemical shift at 39.21 ppm. The C4, C5, C6, and C7 (CH_2_ groups), also secondary carbons, showed chemical shifts ranging from 33.86 to 22.61 ppm. C8 (CH_3_ group), a primary carbon, was identified at 14.09 ppm.

The obtained chemical shifts aligned with those previously reported for the 3-hydroxyoctanoate monomer [44,51]. The ^1^H and ^13^C NMR analyses thus confirmed the presence of a P(3HO) homopolymer in the extracted material, which was synthesized using octanoate as the carbon source. The chemical structure of the P(3HO) homopolymer is illustrated for all types of extractions performed, showing no presence of contaminants (Figure 7 and Figure S4).

## 4. Discussion

The extraction and purification processes for polyhydroxyalkanoates account for approximately 30% of the total production cost, directly influencing polymer quality, end-use applications, and market value [1,10]. Most existing methods rely heavily on chloroform and other organic solvents, which pose significant health and environmental risks. Thus, optimizing these processes is deemed crucial for enhancing the sustainability, economic feasibility, and safety of industrial PHA production [52].

In this study, several extraction methods were evaluated, with their respective recovery yields calculated:

Physical methods: Boiling and sonication yielded considerable recovery percentages, with the former showing slightly higher results (Figure 1 and Figure 2). These methods leverage the resuspension of dried biomass in water, which facilitates PHA separation by centrifugation due to its hydrophobic nature and higher density compared to water [20]. However, differences in yield could be attributed to ultrasonic waves, which destabilize PHAs by creating microbubbles capable of disrupting hydrophobic interactions or even breaking ester bonds within the polymer [37]. This could lead to the loss of PHA-containing carbonosomes and reduced cleanliness of cellular extracts [33,34]. Suboptimal sonicating intensities and durations likely affected the performance for *Pseudomonas putida* U [37].

Chloroform extraction: Despite its low yield per 100 g of biomass (Figure 1), this method achieved approximately 80% extraction efficiency and produced the cleanest extracts with minimal polar contamination (Figure 2). However, further purification steps are required to quantify the actual PHA content due to residual cell components [34,53].

Sodium hypochlorite (NaClO): Extractions using NaClO exceeded 80% efficiency (Figure 1) but resulted in low PHA recoveries (<10% at 90 °C, 20–25% at room temperature) (Figure 2). The oxidative properties of NaClO likely caused significant degradation of the PHAs and cellular structures, highlighting its limited standalone utility for PHA extraction [33,34,35,54].

Alkaline lysis with NaOH: Using 0.01 M NaOH, extraction efficiencies ranged from 70 to 80% (Figure 1), with lower yields observed with extended incubation times [31]. Higher NaOH concentrations (e.g., 0.1 M) were less effective, likely due to polymer degradation induced by elevated alkalinity and temperature [29,31,34]. Nevertheless, this method produced highly purified extracts, particularly under conditions of 0.05 M NaOH for 1 h at 60 °C (Figure 2) [31].

SDS lysis: This method achieved approximately 60% efficiency, comparable to physical methods, but lower than the 80% reported by Mannina et al. (2019) [34] (Figure 1 and Figure 2). As a surfactant, SDS does not significantly disrupt PHA integrity; however, its efficacy depends on the lipid-to-protein ratio of the PHA surface layer, which varies by microorganism [35,55].

Post-extraction analyses using IR and TGA were performed to provide information on the quality of the polymer. Infrared spectroscopy confirmed the presence of characteristic ester bond peaks (C=O and C-O-) (Figure 3). Minor spectral variations in the range of 2500–2300 cm^−1^ were observed, revealing differences in the composition of P3HO, which could be attributed to the CO_2_ present in the samples due to the basic solvents used [56] (Figure 4). The TGA results indicated incomplete volatilization of P3HO at 240–260 °C, suggesting the possible presence of organic and inorganic impurities resistant to high temperatures (Figure 5). Comparative analyses using GPC and NMR showed that the analyzed methods preserved the polymer size and polydispersity index (PDI) as effectively as extraction with chloroform, while minimizing polymer degradation [51] (Figure 6 and Figure 7). Therefore, no significant differences were observed between the tested methods and the chloroform method, as indicated by GPC, which confirmed similar polymer sizes, and NMR, which revealed no detectable impurities in the P3HO.

Collectively, these findings suggest that while chloroform extraction remains reliable, alkaline lysis with NaOH emerges as a promising, sustainable, and cost-effective alternative for extracting P(3HO) from *Pseudomonas putida* U.

## 5. Conclusions

In this study, various methods for the extraction and purification of poly(3-hydroxyoctanoate) (P3HO) produced by *Pseudomonas putida U* were evaluated, focusing on their efficiency, sustainability, and industrial feasibility. Although chloroform extraction yielded high-purity extracts with minimal polar contamination, its reliance on toxic halogenated solvents limits its use in sustainable industrial processes due to the high costs associated with its recycling, cleaning, and safety measures. Alkaline lysis with NaOH, particularly at a concentration of 0.05 M under controlled conditions of 60 °C for 1 h, was identified as a promising alternative, achieving an extraction efficiency of 70–80% while preserving polymer integrity and producing highly clean extracts. Although the recovery yield was slightly lower than that of chloroform extraction, polymer degradation was minimized, and the environmental and economic impacts associated with organic solvents were reduced. The widespread industrial use of NaOH in applications such as paper manufacturing, water treatment, textiles, and food processing supports the scalability of this method. However, some limitations were recognized for large-scale implementation in P3HO production, and additional purification steps would be necessary for applications in medical, food, and health-related sectors to meet strict quality and safety standards. Thus, alkaline lysis with NaOH represents a scalable, sustainable, and economically viable method for P3HO extraction, particularly for industrial applications in markets prioritizing environmental sustainability, though further process optimization and purification steps are recommended for specialized applications.

## Figures and Tables

**Table 1:** Comparison of PDI, Mw, and Mn values of polymers extracted with the different methods. Mw = weight-average molecular weight; Mn = number-average molecular weight; n = polydispersity index (Mw/Mn).

Sample	Mn	Mw	n
Sonication	87,251	157,164	1.80
Chloroform	70,727	125,312	1.77
NaOH 0.01 M, 60 °C 1 h	70,511	123,527	1.75
NaOH 0.05 M, 60 °C 1 h	86,655	154,841	1.79
NaOH 0.1 M, 60 °C 1 h	77,158	142,392	1.85
Boiled	66,560	107,956	1.62
SDS	85,921	151,919	1.77
NaClO	80,184	147,795	1.84

## Data Availability

The original contributions presented in this study are included in the article/Supplementary Material. Further inquiries can be directed to the corresponding author.

## Supplementary Materials

The following supporting information can be downloaded at https://www.mdpi.com/article/10.3390/polym17020150/s1: Figure S1: Extraction of PHA by various methods; Figure S2: Purity of PHA obtained by different extraction; Figure S3: FT-IR of all PHA obtained from the extraction processes; Figure S4: ^13^C NMR spectra of the extracted P3HO. Table S1: Weight percentages of residues identified at 380 °C and 500 °C.
