# Supplementary material for: The Effectiveness of Polyhydroxyalkanoate (PHA) Extraction Methods in Gram-Negative Pseudomonas putida U
Source: Polymers (Basel). 2025 Jan 9;17(2):150. doi: 10.3390/polym17020150 (PMC11769110; doi:10.3390/polym17020150)
Supplement: Supplementary file 1 [file polymers-17-00150-s001.zip › polymers-3410523-supplementary.pdf]

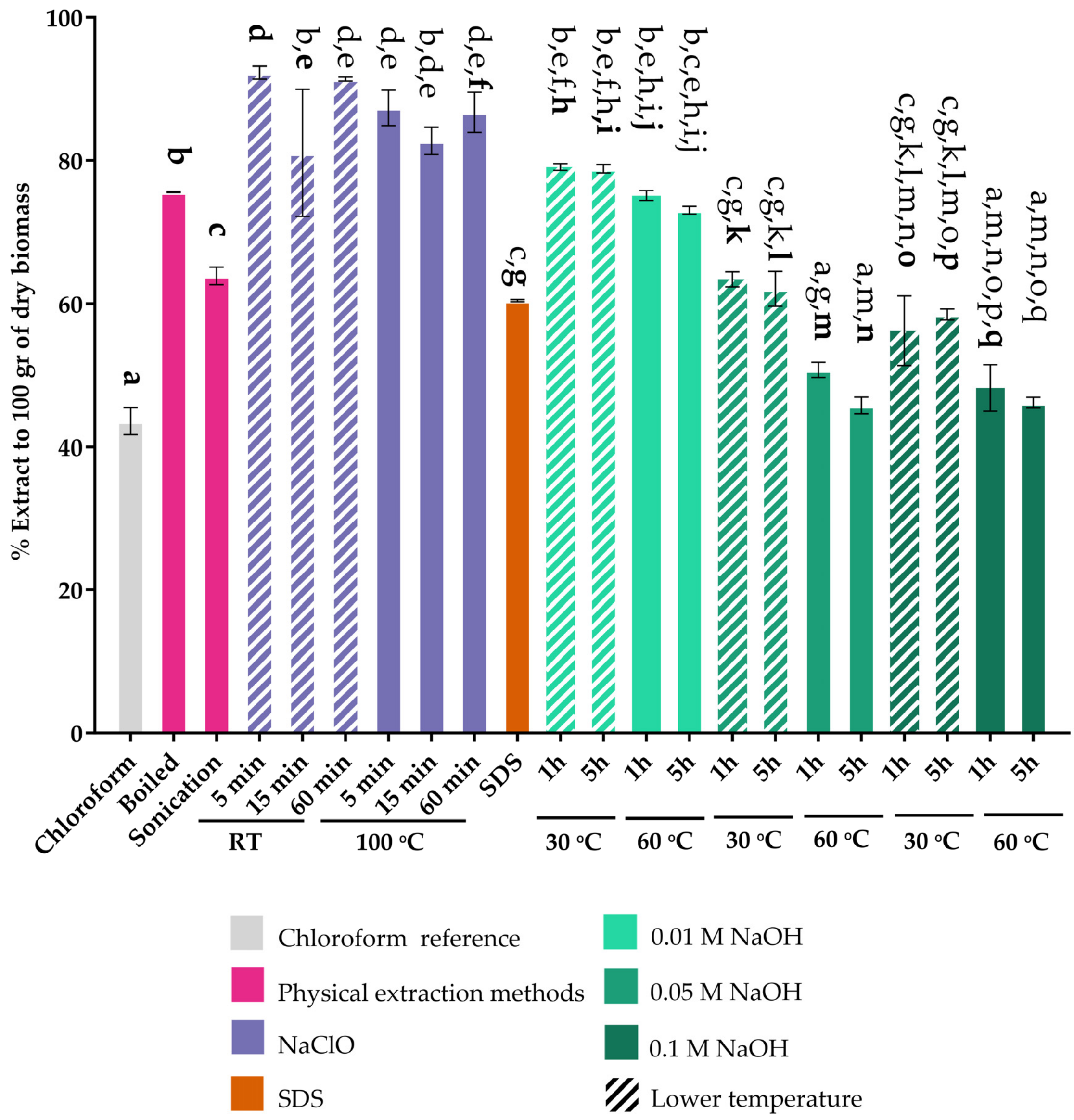

Figure S1: Extraction of PHA by various methods. The chloroform (grey) reference was compared to physical extraction (magenta), NaClO (purple), SDS (orange), and NaOH (green) methods. All samples were run in triplicate and statistical significance was determined with ANOVA between samples ($p \leq 0.05$). The letters present in multiple columns indicate that no significant difference exists compared to the treatment with the same bolded letter.

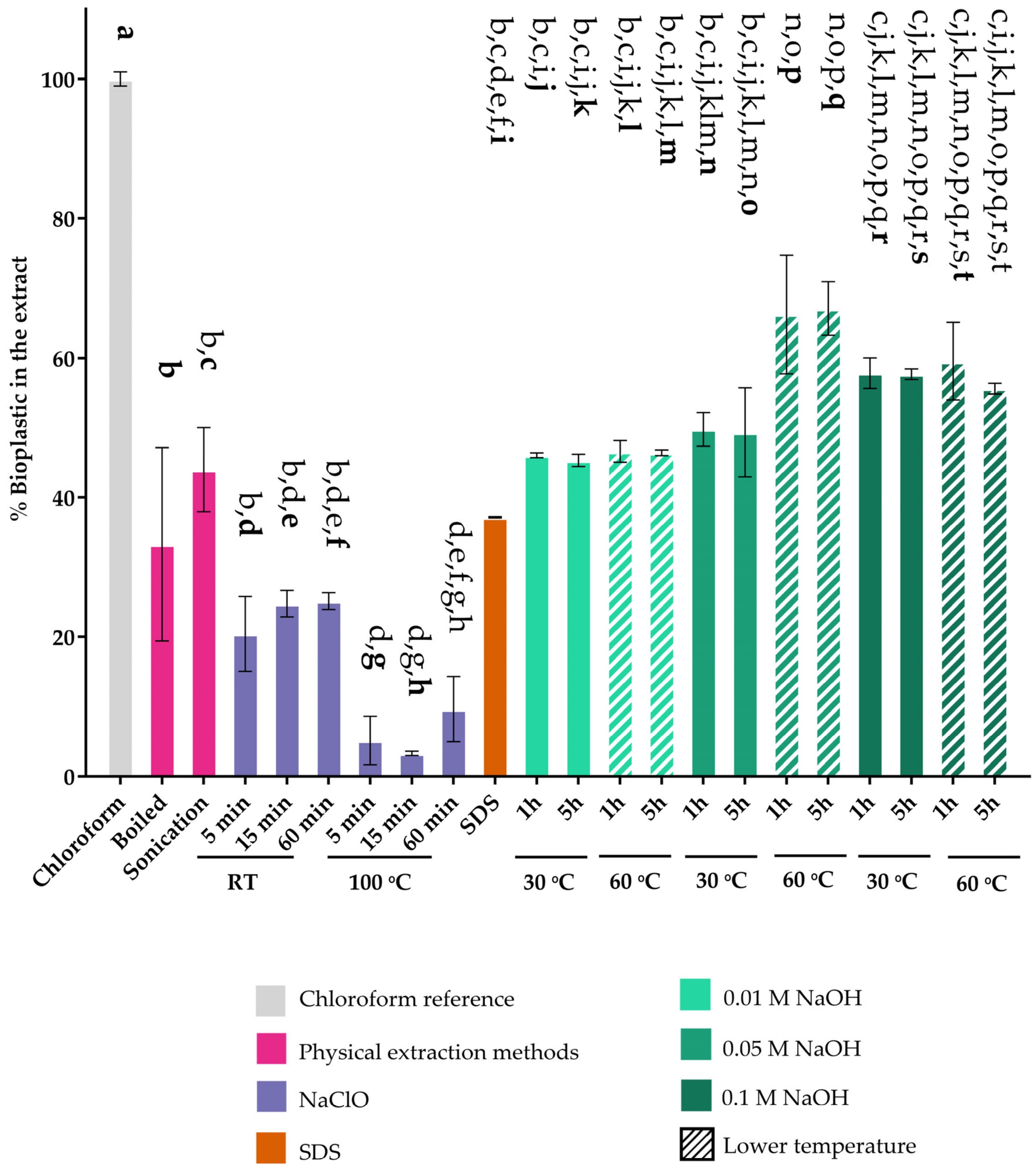

Figures S2: Purity of PHA obtained by different extractions. In grey, we have chloroform as a reference. In magenta the physical treatments are shown, in purple color the hypochlorite treatments, in orange color the SDS treatment and finally in green color the NaOH treatments. All extraction processes gave significant differences with respect to chloroform. Replicate samples n = 3. Statistical significance was determined with ANOVA between samples with respect to chloroform treatment and was not significant for ($p \leq 0.05$). The letters present in multiple columns indicate that no significant difference exists compared to the treatment with the same bolded letter.

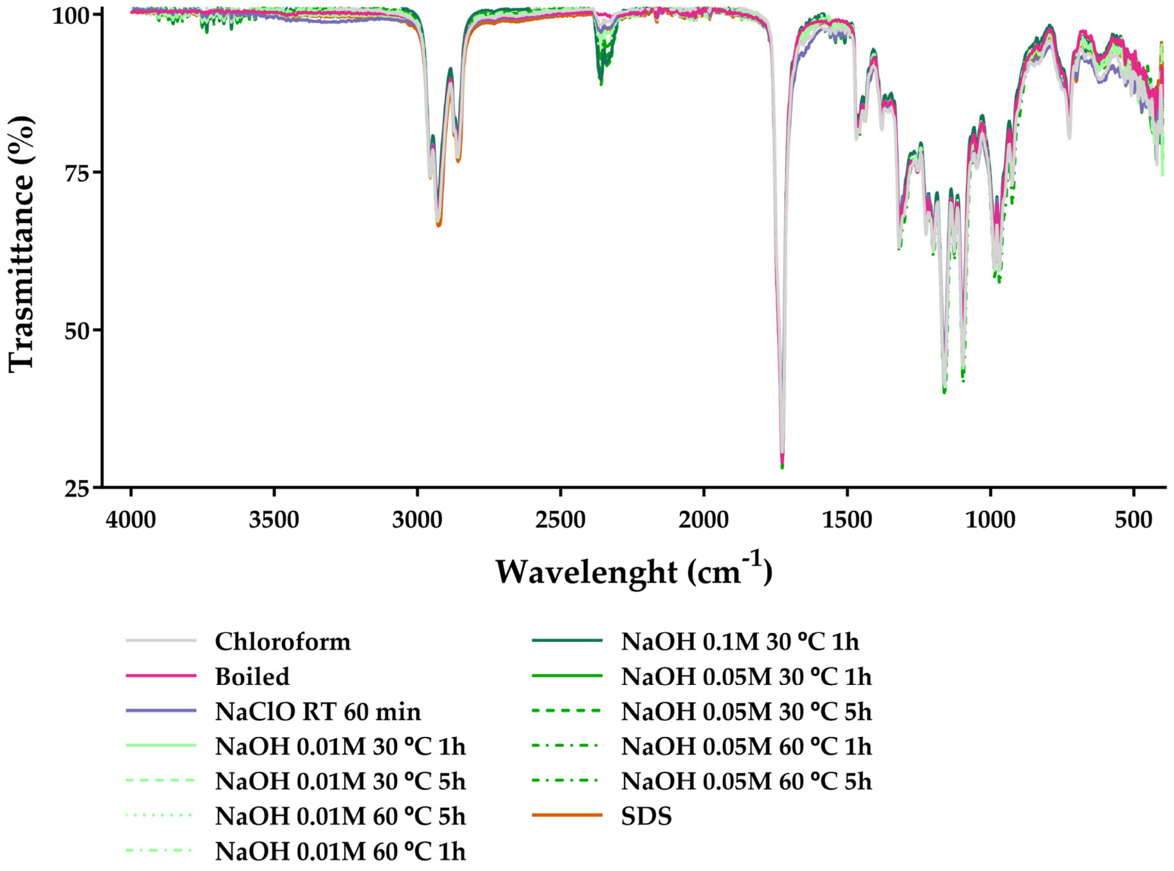

Figure S3. Fourier transform infrared spectroscopy (FT-IR) of the PHA obtained by all the extraction processes where bioplastics were obtained.

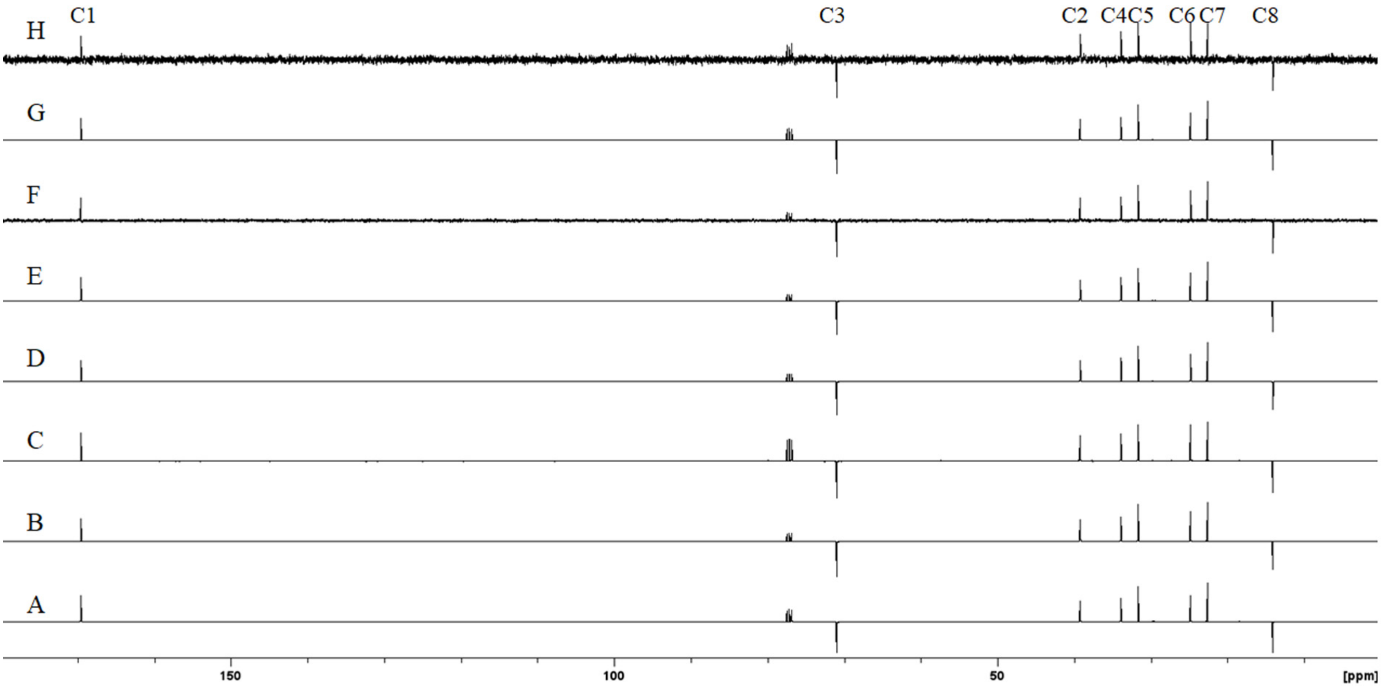

Figure S4. NMR spectra of the P3HO homopolymer extracted using various methods from *P. putida* U. The ^{13}C NMR spectra for the extraction methods are presented below: A) boiling, B) sonication, C) SDS, D) NaClO, E) 0.01 M NaOH, F) 0.05 M NaOH, G) 0.1 M NaOH and H) chloroform. Peaks are assigned to the corresponding carbon positions of octanoate, as depicted in Figure 1. The upward peaks indicate secondary or quaternary carbons, while the downward peaks indicate primary or tertiary carbons.

Table S1. Weight percentages of residues identified at 380 °C and 500 °C.

Samples	Weight at 380 °C (%)	Weight at 500 °C (%)
**Chloroform**	8.7	2.4
**NaClO**	6.2	2.9
**NaOH 0,1M 60 °C 1h**	0.7	< 0.1
**NaOH 0,05M 60 °C 1h**	3.3	0.4
**Other samples**	<0.1	< 0.1
